# The potential of zebrafish as drug discovery research tool in immune-mediated inflammatory disease

**DOI:** 10.1007/s10787-024-01511-1

**Published:** 2024-06-26

**Authors:** Carine Smith

**Affiliations:** Experimental Medicine Group, Department of Medicine, Stellenbosch University, Parow, South Africa

**Keywords:** Disease modelling, Therapeutic target identification, Adverse effects, Individualised medicine

## Abstract

Immune-mediated inflammatory disease (IMID) prevalence is estimated at 3–7% for Westernised populations, with annual incidence reported at almost 1 in 100 people globally. More recently, drug discovery approaches have been evolving towards more targeted therapies with an improved long-term safety profile, while the requirement for individualisation of medicine in complex conditions such as IMIDs, is acknowledged. However, existing preclinical models—such as cellular and in vivo mammalian models—are not ideal for modern drug discovery model requirements, such as real-time in vivo visualisation of drug effects, logistically feasible safety assessment over the course of a lifetime, or dynamic assessment of physiological changes during disease development. Zebrafish share high homology with humans in terms of proteins and disease-causing genes, with high conservation of physiological processes at organ, tissue, cellular and molecular level. These and other unique attributes, such as high fecundity, relative transparency and ease of genetic manipulation, positions zebrafish as the next major role player in IMID drug discovery. This review provides a brief overview of the suitability of this organism as model for human inflammatory disease and summarises the range of approaches used in zebrafish-based drug discovery research. Strengths and limitations of zebrafish as model organism, as well as important considerations in research study design, are discussed. Finally, under-utilised avenues for investigation in the IMID context are highlighted.

## Introduction

After three decades of data collection, the Global Burden of Disease (GBD) study recently calculated the incidence of immune-mediated inflammatory diseases (IMIDs) at almost 1% of the global population (Wu and GBD 2019), with most prevalent IMIDs identified as rheumatoid arthritis, atopic dermatitis, asthma, multiple sclerosis, psoriasis and inflammatory bowel disease (IBD). In addition, prevalence of 3–7% has been estimated for Westernised populations (El-Gabalawy et al. 2010). Potentially the most significant of all statistics on IMIDs, is that all sources seem to agree that IMID incidence is steadily increasing, with rheumatoid arthritis topping the list. Thus, as stated by the GBD group of collaborators in a recent review (Wu and GBD 2019), prevention and integrative management of IMIDs should be prioritised.

In terms of therapeutics, a recent review presented the evolution of IMID treatments, illustrating how progress have been made from broad-spectrum immune modulators such as methotrexate, to highly specific therapeutics targeting among other, cytokines and cytokine receptors, as well as cell receptors (McInnes and Gravallese 2021), in a quest to curb longer-term adverse outcomes. Unfortunately, current commonly prescribed mainstream pharmaceuticals have been estimated on average to have a mere 1 in 25 therapeutic success rate (Schork 2015), clearly indicating the requirement for at least some degree of individualisation in drug discovery approaches. Furthermore, in the context of diseases requiring long-term management, such as IMID, the duration of efficacy testing research trials is generally too short to enable accurate estimation of longer-term drug-associated adverse outcomes. This further complicates long-term patient management and prevention of disease complications, such as heart and kidney disease, which contributes to most rheumatic disease-related deaths.

Given the complexity of chronic diseases such as IMIDs, as well as confounding factors such as genetics, ethnicity (and the Caucasian bias in clinical trials), sex, age, diet, etc. which affects drug efficacy (and toxicity) at individual level (Sun et al. 2022), it is unlikely that current IMID drug discovery approaches will ever yield a magic bullet treatment. Thus, given the immense expense associated with longer-term, longitudinal clinical trials and sufficiently powered preclinical studies in rodents—as well as the generally poor translation of in vitro data (Seyhan 2019)—a new research approach for modern drug discovery in the IMID context is urgently needed.

Literature suggests that zebrafish (both larval and adult forms) could be employed as research tool to fill the gap in this context. This review provides an overview of the appropriateness of the species as human model and provide a review of the most pertinent literature to illustrate how zebrafish may be used as versatile research tool in IMID-related drug discovery.

## Why zebrafish?

Zebrafish (*Danio rerio*) are tropical freshwater, teleost fish that may be housed at relatively high density and significantly lower cost when compared to rodents. Their increased recognition as research tool is evident from the significant increase in their use in published research over recent years (Patton and Tobin 2019). The zebrafish genome has been mapped in its entirety by 2013 and revealed that approximately 70% of human genes have a distinct zebrafish orthologue (Howe et al. 2013), as has 82% of human disease-causing genes (Strynatka et al. 2018), allowing for accurate modelling of the genetic landscape in disease, via genetic manipulation. This high homology with human proteins also accommodates the modern trends in IMID drug discovery, as it allows for targeted manipulation of very specific cellular components, with high translational accuracy. In addition, their high transparency (natural in early larvae, but a genetic modification in adults) lends itself to real-time observation of physiological processes and response to treatments, while their external embryonic development and average lifespan of maximum 3 years (Kishi et al. 2009) allow for investigations (and treatment interventions) spanning an entire lifetime. More than 20 years of drug screening research in zebrafish has demonstrated how therapeutic targets discovered in zebrafish, are highly conserved in humans, as are many pharmacokinetic properties (Patton et al. 2021). Potentially one of the greatest advantages of zebrafish as model, is the speed at which high-throughput screening equipment may be utilised for candidate drug screening (Hall et al. 2014a; b). Furthermore, zebrafish offers the unique opportunity to evaluate drug effects at single cell resolution within a living organism with intact organ systems—given the systemic, multi-system nature of IMIDs, this is a crucial advantage in drug development.

Zebrafish may be used for drug discovery research both in their larval stage and as adults. In the next section, a brief overview of immune system homology is provided, highlighting some benefits and limitations of the model in the context of drug discovery, which should be considered in IMID research study design.

### Zebrafish immune system: benefits and limitations

Regulatory systems are generally highly conserved across species, and particularly so in humans vs zebrafish. In terms of immune system development, myeloid and erythroid cells can already be detected in zebrafish larvae at 12 h post fertilisation (hpf) and definitive haematopoiesis is present from approximately 30 hpf (Jaggannathan-Bogdan and Zon 2013). For the first three weeks of life, zebrafish larvae pose an excellent, clean model for the study of innate immunity (inflammation, natural killer-like cells, complement and interferon) in isolation; then, from approximately 3 weeks of age, zebrafish have fully functional adaptive immune systems with highly conserved functionality in comparison to those of adult humans (Lam et al. 2004).

Due to high gene and protein homology, antibodies raised against human antigens are effective in labelling corresponding zebrafish antigens in the majority of cases (although not all), which reduces the (reagent) cost of preclinical to clinical translation of research data. In support of this, immunofluorescent labelling using anti-human cytokine antibodies and were recently employed in larval zebrafish to illustrate similarities and differences in the acute inflammatory cytokine response between zebrafish and mammals (Ollewagen et al. 2023). Briefly, similar trajectories over time were reported for inflammatory cytokines (MIF, IL-1β, IL-6 and IL-10), but TNF-α and MCP-1 levels remained elevated in zebrafish when those in humans had already normalised. This is likely due to the roles that these cytokines play in tissue regeneration in zebrafish and points out that care should be taken to characterise zebrafish models for purpose before use, to ensure selection of appropriately specific and accurate biomarkers. These data also align with the injury/regeneration literature (Li et al. 2021), which indicates that the inflammatory cytokine response in zebrafish progresses and resolves over a much shorter period, with cytokine levels normalising already at 24 h post-insult, when compared to the same outcome only occurring at 5 days post-insult in mammalian (human and rodent) models (Ollewagen et al. 2023). This is not a limitation per se but indicates that time points for sample collection may be relatively earlier than in mammalian models.

Of specific interest to chronic inflammatory disease, the emerging paradigm that human resolution of inflammation does not rely on neutrophil apoptosis alone, but also their “reverse migration” away from the site of inflammation (Uller et al. 2006), has also been demonstrated in larval zebrafish, using transgenic zebrafish larvae with fluorescent neutrophils and time-lapse microscopy (Mathias et al. 2006), again illustrating the high conservation of innate immune mechanisms across species. In addition, roles of important signalling role players have also been elucidated in zebrafish larvae. For example, using transgenic zebrafish expressing photoconvertible pigments in myeloid cells, hypoxia-inducible factor—which is known to trigger inflammation—was also demonstrated to inhibit neutrophil reverse migration in zebrafish (Elks et al. 2011). These similarities highlight the capacity for parallel studies in zebrafish and humans, using zebrafish to expand on human studies by elucidation of mechanisms at play, which may contribute substantially to therapeutic target identification and/or refinement.

In terms of research focussing on the adaptive system, the sites of maturation of lymphocytes are an important consideration. Early lymphopoiesis takes place in the pronephros (the larval zebrafish kidney, which also fulfils the hematopoietic role of the human bone marrow), with B cells maturing in the zebrafish pancreas (Danilova and Steiner 2002). However, since zebrafish possess bilateral thymi resembling the mammalian thymus, zebrafish T cells mature in the thymi and can also be found—similar to humans—in the spleen, mesonephros (adult zebrafish kidney, as bone marrow equivalent) and epithelial tissue such as gut and nasal mucosa (Langenau et al. 2004). Of further significance, zebrafish do not seem to have lymph nodes. Despite these differences, zebrafish exhibit similar T and B cell maturation, differentiation and responsiveness to stimuli (Liu et al. 2017). Furthermore, of relevance to antibody-mediated immunity, the presence of memory T and B cells in zebrafish has been suggested, while presence of enzyme and gene systems required for developmental programming of B and T cell receptor diversity, as well as diversity in terms of antibodies, has been established (Bailone et al. 2020). Indeed, presence of various immunoglobulin families (Ig) and subtypes (including IgM, but not IgA, IgG or IgE) with diverse functionality has been illustrated using flow cytometry of kidney marrow, in situ hybridisation, gene expression and transgenic fluorescent reporter lines (Fillatreau et al. 2013; Ji et al. 2021; Muire et al. 2017; Wcisel et al. 2023). The plethora of data already generated to characterise the zebrafish adaptive immune system, both facilitates and validates the suitability of using this model in disease modelling and drug discovery in the context of IMID. However, on a somewhat cautionary note, to accurately interpret the significance of immune cell detection in tissue, the altered hematopoietic organ functionality should be kept in mind when modelling IMIDs and IMID-associated complications where these organs are involved, e.g. auto-immune disease-associated kidney disease. However, this potential confounding factor may be overcome with the use of appropriate controls.

### Requirement to also consider other regulatory systems

Part of the complexity of IMIDs is no doubt owing to the involvement of not only the immune system, but also dysregulation of other regulatory systems. Neuroendocrine regulation of IMID is well described (Sternberg 2001) and occurs at different levels to achieve different outcomes (e.g. localised glucocorticoid activation of thymal immune activity vs its systemic anti-inflammatory effect following activation of the hypothalamic–pituitary–adrenal axis). Furthermore, oestrogens play a particularly significant role in the female bias observed in auto-immune and inflammatory diseases. It is therefore important to take a holistic approach in drug discovery in the IMID context especially.

In the endocrine system, in contrast to rodent endocrinology—where the major glucocorticoid is corticosterone, and where significant species differences are present in terms of the sites of steroidogenesis, as well as the cytochrome P450-regulated steroidogenesis pathway itself (Powrie and Smith 2018)—zebrafish also produce cortisol as main glucocorticoid in response to a variety of stressors and their glucocorticoid receptor (GR) genes are highly homologous to those of humans (Schaaf et al. 2008; Dinarello et al. 2020). In fact, zebrafish are the only non-primate animal model in which a GRβ variant has been identified, making it uniquely suitable for the study of GC resistance (Schoonheim et al. 2010).

One difference between zebrafish and humans which may be an important consideration in drug discovery where ion balance, blood pressure and/or fluid volume is manipulated, is that zebrafish do not produce aldosterone, as they maintain ion balance via direct exchange with their aqueous environment (Baker 2019). However, modern IMID-addressing drugs are unlikely to primarily target secondary symptoms such as hypertension. Thus, this difference should not invalidate zebrafish as research model in the current context.

Given the female bias of inflammatory diseases in general, sexual development/maturity of zebrafish in experiments is an important consideration. Zebrafish sex is determined by various genes (i.e. not a single sex chromosome), as well as environmental factors such as temperature, stocking density and food abundance, so that sex is indiscernible prior to sexual maturation at approximately 3 months (Pan et al. 2022). However, this is potentially a benefit in drug discovery, as the oestrus cycle is not a confounder across individuals in this model this early in life. Instead, the role of the female sex may be experimentally simulated in a more standardised manner, by enrichment of embryo media using exogenous sex hormones. This approach also allows for evaluation of potential drug-hormone interaction, which may play an important role in eliminating the male-bias in research which predominated until very recently (Holdcroft 2007). For example, oestradiol enrichment was recently applied in the larval zebrafish model of trinitrobenzene sulphonic acid (TNBS)-induced intestinal inflammation (a recognised model of IBD in both zebrafish and rodents) (Pretorius and Smith 2023a). In this study, the addition of oestradiol at doses equivalent to human (female) gut oestradiol levels, illustrated interactive effects between oestradiol and constituents of the gut microbial secretome products, which affected gut epithelial dysregulation, suggesting the requirement for different dosing strategies—or even mechanisms of action—for IBD-aimed drugs in males vs females.

In terms of the zebrafish nervous system, general circuits, cellular role players and neurotransmitter signalling are similar to that of humans, despite the absence of a distinct prefrontal cortex. One particularly relevant aspect is the significantly more complex trace aminergic system of zebrafish. Zebrafish possess 112 trace amine-associated receptors (TAARs), when compared to only 15 and 6 functional TAARs in mice and humans, respectively (Guo et al. 2022). While trace amine and TAAR research is still in its infancy, the close resemblance of trace amines to neurotransmitters should be considered when modelling disease with a strong neuroregulatory component. However, again this does not invalidate zebrafish for use in IMID drug discovery—in fact, zebrafish studies have contributed significantly to drug discovery in the context of multiple sclerosis-associated demyelination (more on this later). Another consideration of importance to larval models, is that these are underdeveloped organisms that are developing at significant rate. For example, the zebrafish larval blood–brain barrier, consisting of astrocytes, endothelial cells and pericytes, is functional from approximately 3 dpf, but continues to become more effective with further development (Quiñonez-Silvero et al. 2020). Thus, knowledge of the optimal developmental age and standardisation of age at sampling time points are extremely important. Failure to minimise these confounders, may lead to misinterpretation of neuroactivity of candidate drugs, overestimation of neurotoxicity risk, etc., which may lead to inappropriate decisions made in terms of therapeutic potential, bioavailability and safety of candidate drugs.

Another nervous system-related topic of particular interest both in IMID is pain, which has been identified as major contributor to poor mental health outcome in over 40% of a large (*n* = 1196) cohort of individuals suffering from rheumatoid arthritis, inflammatory bowel disease or multiple sclerosis (Almweisheer et al. 2023). In the zebrafish literature, largely due to ethical considerations, much research has been done to understand how (and if) zebrafish are able to experience pain. Zebrafish brain anatomy—and particularly the amygdaloid complex—has been described in detail down to molecular level (Porter and Mueller 2020), with evidence illustrating zebrafish to have nociception systems very similar to that of humans, indeed suggesting that zebrafish—even during larval stages—are able to both perceive and process pain. In the niche of analgesic development for example, zebrafish have long been known as suitable models for the study of opioid activity, as their opioid receptors and associated peptides are fundamentally similar to those of mammals in terms of pharmacological, biochemical and molecular profiles (Gonzalez-Nunez and Rodríguez 2009). Furthermore, pain behaviour in zebrafish (decreased and/or abnormal activity, guarding behaviour, increased behaviour, increased ventilation, deviation in body axis—compared to human hunching) resembles that of humans and has been shown to be normalised after administration of pain-relieving pharmaceuticals (Sneddon 2019). Several models of pain exist in zebrafish. These range from easily executed basic models employing temperature, pH, chemical or mechanical stimuli, to highly advanced electrophysiological models incorporating calcium-sensors and optogenetics, as recently reviewed (Ohnesorge et al. 2021). In terms of specific analgesic classes, anaesthetics such as lidocaine, acetylsalicylic acid have all been demonstrated to have analgesic effects in zebrafish pain models (Lopez-Luna et al. 2017; Costa et al. 2019), confirming the feasibility of extrapolation of effects in zebrafish to humans. However, in the context of NSAIDs in particular, some limitations have been reported, with larvae seemingly less sensitive to reflect therapeutic effects than adult zebrafish (Curtright et al 2015; Schroeder and Sneddon 2017). In the context of NSAID-based drug development, it is thus advisable to include investigations in both larval and adult zebrafish models.

Recently, much attention has also been paid to the role of the microbiome as additional regulatory system in disease, potential mediator of drug resistance and even as candidate therapeutics. While significant inter-species variation in composition of the microbiome exists, zebrafish may be suitable for microbiome-relevant research. Zebrafish larvae only establish their own microbiome after development-linked opening of the snout and anus (after 4dpf), and then acquire a microbial structure similar to that of humans (i.e. dominated by *Firmicutes* and *Bacteroides*) (Zhong et al. 2022; Xia et al. 2022). In the context of IBD, several trace amines (as microbial secretome constituents) have been successfully screened for their role in disease outcome, with some illustrated to exacerbate disease, while others may alleviate inflammatory outcome (Pretorius and Smith 2023a).

In summary, zebrafish physiology compares very favourably with other preclinical models in terms of similarity to human physiology. However, as with any model of human disease, it is important to remember that it remains a model—familiarisation with relevant systems and physiological processes is key to successful use of this extremely versatile organism. In the section below, the unique versatility of zebrafish in terms of research methodology is highlighted.

### Unique possibilities for data generation

From the sections above, it is clear that many commonly used analytical techniques such as flow cytometry, immunohistochemistry, microarrays, -omics, optogenetics, etc., are equally viable options for data generation in zebrafish. In addition, zebrafish present some additional unique opportunities for creative study design and data collection.

For example, the use of transgenic zebrafish to label specific immune cells/cell components and signalling molecules (including cytokines) described above, has become a popular approach for visualisation of cell migration and accumulation, cytokine signalling, etc. in real time (including time-lapse) and in living larvae. Although generation of transgenic animals is a somewhat time-consuming process, requiring validation of transgenic success only in the F3 generation, the relatively fast sexual maturation and high fecundity of zebrafish makes this a popular option. In addition, gold standard transgenic fluorescent reporter lines are widely available and accessible, so that specific genetic knowledge is not a prerequisite for their use. However, it is important to ensure adequate specificity of the label used, for the intended purpose. For example, transgenic labelling of *mpeg 1* (coding for perforin-2 on macrophages) is commonly used to label zebrafish larval macrophages but has recently been shown to also label a subpopulation of B cells in adult zebrafish (Ferrero et al. 2020), suggesting that not all parameters “are equal” across adult and larval models. Another potential drawback of this technology is that most transgenics are labelled using either green fluorescent protein (GFP) or mCherry (red)—although hybrids of different transgenics may be prepared to achieve simultaneous assessment of more than one parameter, the number of parameters that can be visualised simultaneously, is limited to the number of fluorophores available. Also, given the relatively large size of fluorophores, smaller molecules cannot be labelled in this way without affecting their functionality.

Another common approach is the use of morpholinos (MO). MO are DNA antisense oligonucleotides which, when injected into embryos between the 1- and 8-cell stage, disrupts normal synthesis of the targeted protein for the duration of MO activity timespan (up to 10 days), creating a temporary knockout model (Trede et al. 2004). Given the limited duration of morpholino activity, this approach is of course only feasible for the study of innate immune mechanisms in larvae. A huge benefit of this approach is the speed at which a gene of interest may be knocked out, whilst retaining the benefit of a whole organism context.

An exciting, but still under-researched, avenue for investigation that is particularly relevant to IMID research, is the potential for xenograft transplantation (Di Franco et al., 2022). Given the absence of the adaptive system in larvae, xenografts are possible without any host preconditioning, although it comes with a significant limitation in terms of feasible xenograft size. However, adult xenograft models have been achieved to circumvent this problem. For example, the cmyb^I181N^ zebrafish strain lacks specific haematopoiesis, which enables xenografts to be transplanted into adult zebrafish without the confounding effect of the host adaptive immune system, enabling the study of species-specific role players in haematopoiesis and the adaptive system in particular (Iwanami et al. 2017). Given the extensive genetic toolbox available in zebrafish, it should be possible to create IMID-relevant xenograft mutants allowing for direct human-specific drug efficacy assessment in models more closely modelling human disease. Furthermore, such a model will allow for an individualised medicine approach, as patient-specific xenografts may be performed, creating individual patient-representing avatars—much like the co-clinical trials employed in oncology research (Usai et al. 2021). On a practical note, despite species differences such as physiological body temperature (28 °C for zebrafish), human monocytes have been shown to survive and differentiate normally in zebrafish (Paul et al. 2019), supporting the validity of a xenograft approach in IMID disease modelling and drug testing.

In term of drug delivery, the transparent zebrafish—especially during the larval stage—provides an excellent platform for in vivo, time-lapse assessment of drug delivery systems in terms of delivery efficacy, drug therapeutic availability, off-target effects and potential toxicity risk. Zebrafish are used extensively for the assessment of nanosized drug delivery and organ distribution across biological barriers, as well as nanotoxicity risk (Li et al. 2017; Jia et al. 2019).

Lastly, the unique capacity to combine quantitative behavioural analysis with physiological responses to drugs and/or drug effects, and the speed at which assessments may be completed, makes the zebrafish model a popular tool for the assessment of toxicity risk—and particularly neurotoxicity risk—of candidate drugs. For example, during the recent SARS CoV-2 pandemic, the antiparasitic drug ivermectin was proposed as feasible anti-viral option, based on positive in vitro results. However, when human equivalent concentrations were assessed for safety in larval zebrafish (Powrie et al. 2022), significant neurotoxicity were elucidated at claimed anti-viral doses. Zebrafish is also emerging as a popular model in the ethnomedicine drug discovery sphere, where standardised larval models may be used to screen for drug efficacy, as well as for relative therapeutic contributions by—and interactions between—the various potential active components within a plant-derived extract (Gericke et al. 2024)—this may significantly refine ethnomedicines going forward.

Given the extensive research methodology toolbox that already exist for zebrafish—in addition to their generally conserved physiology and unique added benefits in terms of fecundity, transparency, live imaging and relatively simple anatomical structure of zebrafish models are already making unique contributions at all stages of drug discovery in the context of immune-mediated inflammatory disease.

### Drug disposition in zebrafish

The relevance of drug absorption, distribution, metabolism, excretion and toxicity (ADMET) characteristics is of vital importance in an animal model used for drug discovery. Drug administration for the purpose of toxicity assessment, is usually performed via immersion of larvae into drug-containing larval maintenance media, which is a buffered aqueous solution containing low concentrations of salts only. It is therefore important to consider lipophilicity of drugs, which may result in low solubility in aqueous vehicles. Of interest, increased uptake of lipophilic drugs has been reported in zebrafish larvae (Poon et al. 2017). Thus, to mimimise these opposing confounding effects on drug uptake and, thus, LD50 determination, it is advised to dissolve lipophilic drugs in suitable solvents such as DMSO or ethanol, before diluting into media, taking care to reduce the final concentration of these solvents to well below reported tolerance levels in zebrafish (Maes et al. 2012). Similarly, it is advisable to ensure that pH of medium remains within ranges tolerated by zebrafish larvae, after addition of low-pH drugs.

Drug distribution assessment in larvae is still a developing field. Although mass spectrometry or HPLC quantification of drug and metabolite levels in different organs or tissues is a possibility when using adult zebrafish, this approach—as in mammalian models—remains expensive and time consuming. In a different approach, using naturally fluorescent chemotherapeutic compounds, tissue-specific accumulation of administered compounds was shown to depend on drug structural profile, and to correspond to clinical pharmacokinetic indexes in most cases (Yao et al. 2017). Although wider validation of this method is still required—including potential confounding effects of fluorescent labels on physiological processes— in vivo visualisation of tissue distribution patterns of drugs in zebrafish larvae as proxy for pharmacokinetic indexes, with the added benefit of elucidating potential for off-target effects, is an enticing angle to pursue.

In terms of drug metabolism and toxicity, an enormous body of evidence shows that drugs known to cause toxicity in humans, elicit similar toxic effects in zebrafish (Jomaa et al. 2014). Zebrafish have been shown to express a full complement of cytochrome P450 (CYP) enzymes. However, while these enzymes share high homology to human CYPs important for endogenous substrate metabolism, xenobiotic metabolising CYPs show more sequence diversity between species (Goldstone et al. 2010), suggesting that at least some drugs may be metabolised into metabolites different from the scenario in human livers. Fortunately, the ease by which zebrafish may be genetically manipulated, allows for a remedy in this instance: generation of a “humanised” zebrafish transgenic expressing human CYP3A4 has been reported (Poon et al. 2017), with which human drug metabolism may be more accurately reflected in zebrafish. Although the added sophistication of this model is probably not a requirement in most instances, it may add significant value in cases where drug metabolites may pose greater risk of toxicity than the parent drug. To evaluate drug-induced renal toxicity, proteinuria may be quantified in zebrafish by assessing protein concentration in maintenance media—with the proviso that confounding factors such as faeces and feeds in media are controlled. An alternative approach to assessment of kidney function is the use of transgenic fish with fluorescent gene reporters for proteins of interest, such as vitamin D-binding protein, which is the zebrafish analogue for albumin (Lim et al. 2022), and which may be used to assess kidney function in vivo or for determination of excreted protein by quantification of fluorescence in media.

In terms of drug excretion, similar approaches as in mammalian models could theoretically be followed, with the caveat that zebrafish do not have a bladder from which urine may be collected. Although methodological advances and high-sensitivity equipment may enable quantification of parent drugs and metabolites excreted into maintenance media in urine or faecal matter, this is not common practise. At this point, zebrafish is probably most suitable for assessing drug uptake, tissue distribution, efficacy and toxicity of drugs, while protocols for assessment of metabolism and excretion still require further development and validation. However, given the large component of drug discovery consisting of drug safety and efficacy testing, as well as therapeutic target identification, the enormous potential of zebrafish as research tool is undeniable.

## Zebrafish approaches in IMID drug discovery

A comprehensive review of general models of inflammation, including role players and useful biomarkers, are beyond the scope of this review, but a recent review (Leiba et al. 2023) is recommended as valuable resource in this context. Instead, this section will review the potential of zebrafish as drug discovery research tool in the context of IMID, focusing on the most prominent IMIDs identified by the GBD study.

### Asthma

The versatility of the zebrafish as research model is perhaps best validated by its use in pulmonology research, despite the absence of lungs in this aquatic species. Significantly, the zebrafish gills function very similarly to human airways (Paik et al. 2015), while the swim bladder (important for buoyancy) closely resembles human lung anatomy (Winata et al. 2009) and cellular morphology, including the presence of epithelial surfactant (Robertson et al. 2014). Furthermore, transcriptomic analysis suggests high molecular homology between the zebrafish swim bladder and human lungs (Zheng et al. 2011).

Asthma is arguably the IMID responsible for highest global disease burden (Wu and GBD 2019). Although it is not possible to induce experimental asthma (bronchoconstriction) in zebrafish, they are an accurate model for the study of specific disease mechanisms, such as the mechanisms for transgenerational inheritance of epigenetic risk factors (Hammer et al. 2018), or the interplay between innate immune cells and the respiratory epithelium (Progatzky et al. 2016). In this context, an accepted, standardised model for inducing chronic respiratory epithelial inflammation in the zebrafish swim bladder is exposure of fish to extracts from cigarette smoke (a known risk factor linked to asthma) (Hammer et al. 2018). Moreover, a model recently described for the modelling of lung injury, is the folic acid deficient (FD) zebrafish mutant, which mimics lung injury resulting from the known asthma-linked protease/protease receptor imbalance (Lee et al. 2019).

The value of in vivo experimentation in organisms allowing for visualisation of drug effects across organs should not be underestimated, especially in the context of detecting potential for drug repurposing. One such example is terbutaline sulphate—a β_2_-adrenergic receptor agonist commonly used to relax bronchial and vascular smooth muscle in asthma. Studies in zebrafish illustrated both preventative and therapeutic effect of terbutaline sulphate in the context of axon defects and neuromuscular degeneration (Paik et al. 2015). Using co-treatment with a β_2_-adrenergic receptor antagonist, β_2_-adrenergic receptor agonism was shown to be responsible for this effect, which may offer benefit to patients with related neurological pathology, such as amyotrophic lateral sclerosis or multiple sclerosis.

### Atopic dermatitis

Atopic dermatitis is another high-prevalence IMID and although not life-threatening, may significantly affect quality of life. The condition is thought to have at least some hereditary risk, as well as immune system hypersensitivity. Zebrafish skin already covers embryos by 3 dpf and, although being devoid of sebaceous glands, hair follicles and a stratum corneum, also have epidermal and dermal layers with a conserved epidermal–dermal junction, as well as highly conserved skin cell-specific genes, which favourably positions zebrafish as tool in dermatology research (Frantz & Ceol 2022).

In the context of atopic dermatitis, mechanistic zebrafish models have indeed already contributed to our understanding of disease mechanisms. For example, the role of nicotinamide phosphoribosyltransferase (NAMPT)-derived NAD^+^ in the development of skin inflammation (via hyperactivation of PARP1, to result in parthanatos cell death) was illustrated in the context of psoriasis (Martínez-Morcillo et al. 2021) and atopic dermatitis (Arroyo et al. 2023). A strength of the approach taken by this group, is the parallel assessment and validation of the mechanisms elucidated in zebrafish, using human models (human organotypic 3D skin model of both psoriasis and atopic dermatitis), to achieve immediate translation and proof of relevance. Moreover, the mechanism whereby elevated β-oxidation-fuelled mitochondria-derived reactive oxygen species within epidermal cells was shown to guide matrix metalloproteinase-driven leukocyte recruitment was first demonstrated in zebrafish (Hall et al. 2014). Mechanistic models may also contribute to the development of symptomatic treatment in IMIDs such as atopic dermatitis. For example, in line with the recently reported role for toll-like receptor 7 (TLR7) overexpression in chronic eczema development and progression (Wen et al. 2023), employment of a TLR7 agonist (imiquimod) elicited itch-like behaviour in both larval and adult zebrafish, which was mechanistically linked to activation of nociceptive neurons via the transient receptor potential 1 (TRP1) channels (Esancy et al. 2018). Here again, complimentary data were generated in parallel using zebrafish and rodent models, showing not only the conserved nature of inflammatory disease mechanisms across species, but no doubt also increasing translatability of data jointly generated, into the human context, while reducing the number of sentient animals used in research.

### Psoriasis

Psoriasis is thought to result from inappropriate keratinocyte activation to release pro-inflammatory mediators which in turn recruit neutrophils, macrophages and mast cells, triggering active disease. Zebrafish models have been used for some time now to study psoriasis disease mechanisms and some genetic models—such as the *atp1b1a* and *spint1a* mutants—accurately displays hallmarks of human disease, such as dysregulated keratinocyte differentiation, keratinocyte hyperproliferation and chronic neutrophil skin infiltration (Webb et al. 2008; Carney et al. 2007).

It is important however to make a distinction between zebrafish models capable of accurately simulating human disease and mechanistic models aimed at simulating *selected* pathological processes, and to employ the model most appropriate to provide answers to the research question posed. Mechanistic models, although more limited in terms of holistic disease phenotype, are still extremely useful in mechanistic studies, as they allow for isolation of pathological processes and determination of causality, which can be difficult to tease out using more complex disease models, or human patients, especially where comorbidities are already present. In the current context, the somewhat simpler ultrastructure of zebrafish skin (Martínez-Navarro et al. 2019) may actually be an advantage, as—together with high similarity to human epithelium and inflammatory mediators secreted under stimulated conditions—it facilitates research focused on early-disease aetiology. In larval models—where the adaptive immune system is still absent—the ability to accurate simulate early-disease epithelial pathology, makes the zebrafish models ideal for high-throughput drug screening and for studying the initial triggers of inflammatory disease in relative isolation.

Furthermore, given the ease of genetic manipulation of both genetics and the environment of zebrafish, zebrafish has been named the “newly appointed player” in psoriasis research (Martínez-Navarro et al. 2019), where it may be able to fill the gap in knowledge by elucidating the genetic vs environmental roles in psoriasis aetiology, which may further assist with patient management and/or medicines development. Here again, choice of model is an important consideration. For example, mutant models—such as *pen/lgl2*, *Psoriasis/m14, atp1b1a* and *spint1a* mutants—simulating keratinocyte cellular pathology and disruption of the basal epidermis to model psoriasis disease, may be used to understand the genetic basis of psoriasis. Other models facilitate mechanistic studies to better understand disease aetiology and potentially identify new therapeutic targets or screen for treatment efficacy. One example here is the morphant (knock-down) *tnfa/tnfr2* fish, which inhibits *TNF-α* and its associated receptor, to enhance neutrophil infiltration into the skin. In addition to genetic models, environmental inducible models of psoriasis have also been described, such as the germ free (*GF*) model which allows for experimental elucidation of the role that specific microorganisms may play in affecting disease severity.

### Rheumatoid arthritis

Synovial joints are commonly affected in rheumatoid arthritis (RA) and thus, is a focus in drug discovery. Although ray-finned fish species—which include zebrafish—were thought to lack lubricated joints, zebrafish indeed possess synovial lubricated joints in their jaws and pectoral fins (Askary et al. 2016). The same study also illustrated that mutants lacking the prg4b gene (the homologue to human prg4, or lubricin), exhibited similar age-associated joint degeneration observed in mice and humans with lubricin deficiency. Unfortunately, despite this avenue for creating a RA model in zebrafish, no subsequent studies were found implementing this. However, given the fast increase in global incidence of RA, options such as this one should be revisited and prioritised.

Like rodent models, zebrafish models may be criticised for their differences from humans in terms of posture and skeletal muscle anatomy, which may theoretically affect development and progression of RA complications such as rheumatoid cachexia. However, since rheumatoid cachexia profile is related to time since onset of disease, rather than pain-associated reduced activity (Ollewagen et al. 2021a), these differences are unlikely to be of significance in this context. Rather, the fact that muscle atrophy and weakness appear to be present already early in disease—before pain and joint swelling is reported (Costamagna et al. 2015)—and the fact that chronic inflammatory dysregulation rather than impaired myogenic regenerative potential is the major contributor to muscle atrophy in RA (Ollewagen et al. 2021b), position zebrafish as ideal organism in which to study chronic inflammation-associated cachexia early in disease aetiology and over a lifespan, which is not financially feasible in rodents. Given the highly conserved nature of immune function in zebrafish, the use of this model in RA research and drug discovery should be considered.

Unfortunately, current zebrafish-based drug discovery research related to RA, as well as other rheumatic diseases such as systemic lupus erythematosus (SLE), seem to focus mainly on general anti-inflammatory modalities—which are investigated in the general inflammatory models induced by exposure to lipopolysaccharide, leukotriene B4 or copper (Xie et al. 2021). Disappointingly—as is the case for non-specific in vitro models of inflammation induced by LPS, etc.—these general “screening” models seem to be next in line to be abused by researchers not willing to progress past initial screening, instead functioning in silos and producing relatively “dead-end” proof of efficacy data lacking in clinical translation, which has already been identified as significant limiting factor in translation of preclinical drug development data (Pretorius and Smith 2023b). Thus, in the context of RA and rheumatic disease in general, zebrafish as disease model for drug discovery, is extremely under-utilised.

To advance development of more selectively targeting therapeutics in the RA context, more commitment by researchers to model disease-specific mechanistic pathologies, is required to advance drug discovery via generation of more disease-relevant preclinical data. Some studies are emerging though, which demonstrate the elegance with which zebrafish may be used in rheumatic disease research. For example, using a fluorescent gene reporter for matrix metalloproteinase-2 (MMP-2/gelatinase A), the time course and localisation of MMP-2 activity was profiled during the development of the zebrafish tail (Wyatt and Crawford 2021). By showing how upregulation of MMP-2 correlated with collagen degradation (normal during the development process), this study confirmed the likelihood of a significant role for MMP-2—which is upregulated in the joints of patients with RA (Itoh et al. 2002)—in RA disease progression. Furthermore, in line with evidence from rodents suggesting a role for dysregulated autophagy as contributor to joint degeneration and apoptosis-resistance in RA (Vomero et al. 2018), a CRISPR knockout model in zebrafish was recently used to elucidate the role of autophagy (and its inhibition) in chondrocyte development and joint formation in larval zebrafish (Moss et al. 2021). Given the complexity of roles described for autophagy in maintenance of homeostasis—especially in terms of its regulation of immune processes—the zebrafish model may be an ideal candidate for refinement of treatments aimed at inhibiting autophagy. In particular, the ease of visualisation of responses to intervention in live larvae, may prove useful in simultaneously evaluating risk for adverse effects. However, to date, RA remains largely unexplored in zebrafish.

### Inflammatory bowel disease

IBD is commonly researched in murine models, but these models do not allow for visualisation of pathology prior to death, or for visualisation of disease development over time in live animals (Sundberg et al. 1994; Yang et al. 2014). The similar cellular structures of the human vs zebrafish gut allow for investigations into specific molecular regulatory processes, for example the regulation of intestinal goblet cell maturation via modulation of *agr2* expression (implicated in IBD) by *Foxa2* and *Hif1ab* (Lai et al. 2016). Furthermore, 163 SNPs has already been linked to IBD in GWAS studies (Hanyang et al. 2017); thus, a model allowing for ease of genetic manipulation may contribute considerably to confirmation of the relative significance of these SNPs to IBD aetiology, and for the incorporation of major genetic confounders into models used for drug discovery in this context.

It is not unexpected then that several models for simulating IBD—or more specifically, intestinal inflammation—have been developed in zebrafish, for example using trinitrobenzene sulfonic acid (TNBS) as trigger (Xie et al. 2021). The short-duration protocols employed in larval models has established these models as vital models in high-throughput candidate drug screening exercises (Huang et al. 2021; Yu et al. 2021; Xu et al. 2024). A strength of the zebrafish as drug discovery model, is the ability to directly link functional outcome to drug-induced cellular and molecular modulation. For example, treatment-associated normalisation of the gut epithelial tight junction protein expression and distribution patterns in the TNBS model, was linked to normalisation of superimposed induced diarrhoea- and constipation-like changes in gut motility (Pretorius and Smith 2024) in zebrafish larvae. These experiments also demonstrate the capacity for superimposing more than one disease symptom in the same organism.

Furthermore, the ease of creating germ free (GF) zebrafish—which can survive as GF organisms for up to 30 days in a sterile environment—by surface sterilisation of the egg chorion, facilitates their use for the study of microbiome contributions to IBD aetiology and disease progression (Ni et al. 2023; Kamareddine et al. 2020; Flores et al. 2023). This model is, therefore, commonly used for probiotic drug discovery in this context (Chen et al. 2023; Huang et al. 2021). While the argument may be made that many of these models are somewhat non-specific and, thus, not directly applicable to IBD without corroboration in more complex models, a very recently developed transgenic (*Trmt5*-deficient) zebrafish which spontaneously exhibit IBD symptoms (Zhao et al. 2023), seems to more accurately simulate the complexity of the condition. The stability of the mutation allows for drug discovery research across larval and adult zebrafish, which will facilitate longer-term, longitudinal study design.

### Multiple sclerosis

Multiple sclerosis (MS) is potentially the IMID which has enjoyed most benefit from zebrafish-based research to date. For example, in terms of understanding disease mechanisms, genetic mutations linked to MS via GWAS studies, have been induced in zebrafish to elucidate dysregulated processes such as accumulation of autophagosomes and unhealthy mitochondria during brain development (Smits et al. 2023), confirming the role of specific genetic role players in disease aetiology. In terms of drug discovery specifically, the identification of T cell-modulating small molecules has been reported in zebrafish, which may have application in leukaemia and multiple sclerosis (Trede et al. 2013). Several zebrafish models of MS have been described, as previously reviewed (Burrows et al. 2019). A model of experimental auto-immune encephalitis (EAE) is commonly used in drug discovery to elucidate drug efficacy and/or mechanisms (Sunke et al. 2019; Ye et al. 2015).

The MS niche has also demonstrated the possibility of using humanised zebrafish in drug discovery. For example, a GPR17-humanised zebrafish model was recently validated as accurate drug discovery tool for the study of therapeutic GPR17 modulation to promote re-myelination (Häberlein et al. 2022), suggesting huge potential for the use of humanised zebrafish in drug discovery.

**Fig. 1:**
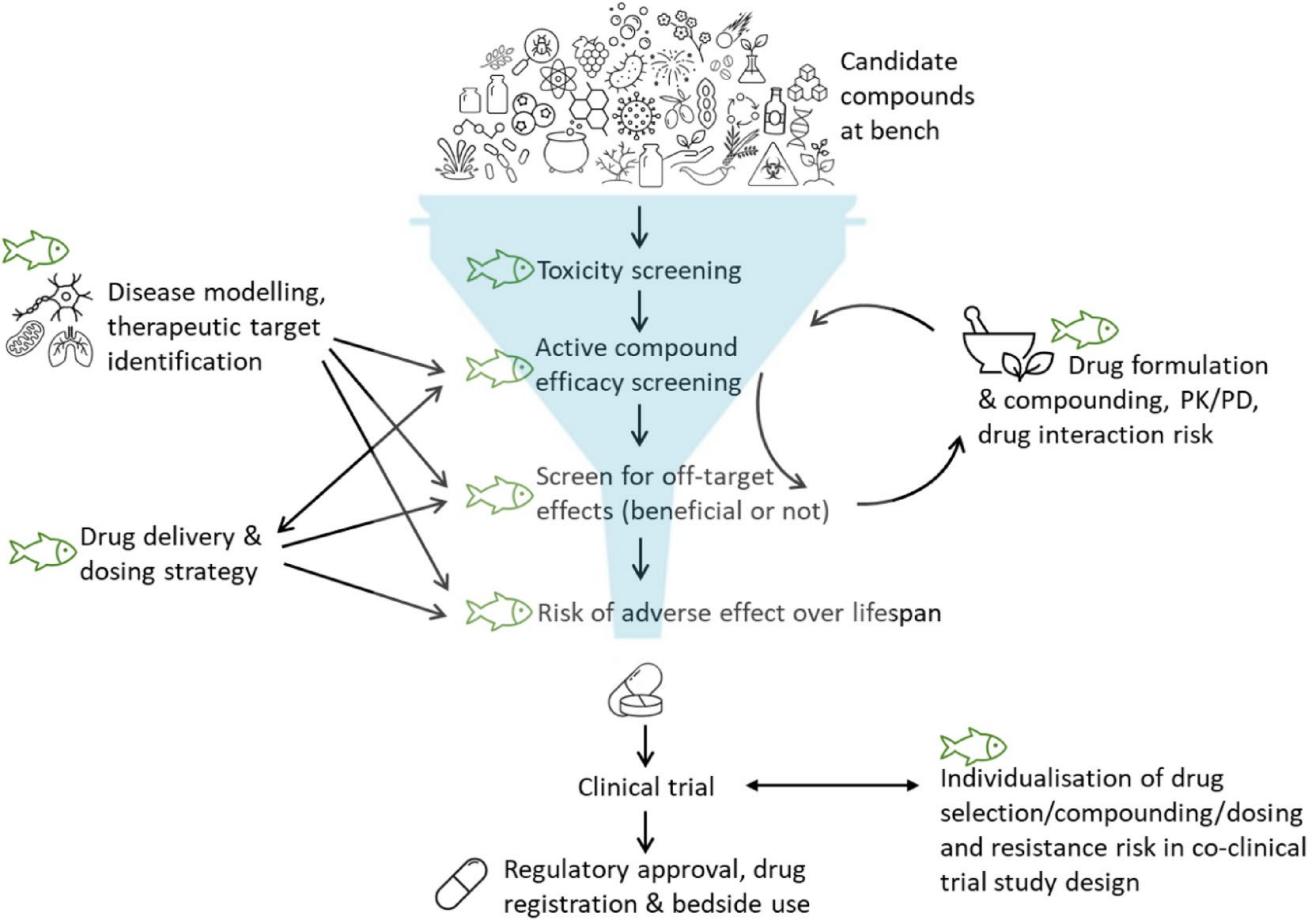
Illustration of the many sectors of the IMID drug discovery pipeline where zebrafish may be used to improve the bench-to-bedside throughput time, efficacy and cost

In summary, from the cited publications, it is clear that, apart from being compatible with common modern research approaches followed in mammalian pre-clinical research, zebrafish as disease model provides high potential for creativity in IMID drug discovery research like never before. Notably, literature indicates that by using zebrafish at all pre-clinical stages of drug discovery (as presented in Fig. 1), this process can be refined to prevent wasting money on candidate drugs with low potential—thereby minimising the financial burden of unsuccessful clinical trials—whist improving on overall candidate drug safety, expanding on capacity for detection of repurposing potential and contributing to overall time economy. Furthermore, although still under-researched in the IMID arena, the potential for individualising medicine by predicting and managing treatment efficacy or resistance in a co-clinical trial-type study design in zebrafish, to account for confounding factors such as genetics, comorbidities, etc., warrants more investment.

## Conclusions

Fifty percent of all deaths globally is ascribed to inflammation-related diseases (Roth and GBD 2017), clearly highlighting that new research approaches to drug discovery in terms of IMID should remain a priority. From the current review, it is clear that numerous zebrafish-based IMID-relevant disease models exist. These are being used—albeit still on relatively small scale—to elucidate new or confirm suspected disease mechanisms, as well as therapeutic targets, in the context of IMID. The fact that zebrafish can easily be genetically manipulated also holds potential for the creation of purpose-specific disease models, for the elucidation of mechanisms of action, drug efficacy and safety over the course of an entire lifespan. The extensive toolboxes that have been developed for research in this organism, positions zebrafish as an invaluable in vivo research tool capable of high-throughput data generation in drug discovery. In this context, in vivo translation of in vitro proof of efficacy data using zebrafish could refine early treatment efficacy screening by balancing physiological relevance and efficacy vs safety. Finally, despite the versatility of this model organism, prioritisation of preclinical data translation is imperative for achieving improved success rate in bench-to-bedside progress, especially in the ethnomedicine sphere (Pretorius and Smith 2023b). Without greater collaboration between bench- and clinician researchers, as well as the manufacturing pharmaceutical industry—at both disease modelling and clinical translation phases—even the best preclinical model will have academic merit only.

## Data Availability

Not applicable, no data sets were generated.

## References

[CR1] Almweisheer S, Bernstein CN, Graff LA, Patten SB, Bolton J, Fisk JD, Hitchon CA, Marriott J, Marrie RA, CIHR Tea in Defining the Burden and Managing the Effects of Immune-mediated Inflammatory Disease (2023) Well-being and flourishing mental health in adults with inflammatory bowel disease, multiple sclerosis and rheumatoid arthritis in Manitoba, Canada: a cross-sectional study. BMJ Open 13(6):e073782. 10.1136/bmjopen-2023-073782 37295825 10.1136/bmjopen-2023-073782 PMC10277148

[CR2] Arroyo AB, Bernal-Carrión M, Cantón-Sandoval J, Cabas I, Corbalán-Vélez R, Martínez-Menchón T, Ferri B, Cayuela ML, García-Moreno D, Mulero V (2023) NAMPT and PARylation are involved in the pathogenesis of atopic dermatitis. Int J Mol Sci 24(9):7992. 10.3390/ijms24097992 37175698 10.3390/ijms24097992 PMC10178103

[CR3] Askary A, Smeeton J, Paul S, Schindler S, Braasch I, Ellis NA, Postlethwait J, Miller CT, Crump JG (2016) Ancient origin of lubricated joints in bony vertebrates. Elife 19(5):e16415. 10.7554/eLife.16415 10.7554/eLife.16415 PMC4951194 27434666

[CR4] Bailone RL, Fukushima HCS, Ventura Fernandes BH, De Aguiar LK, Corrêa T, Janke H, Grejo Setti P, Roça RO, Borra RC (2020) Zebrafish as an alternative animal model in human and animal vaccination research. Lab Anim Res 7(36):13. 10.1186/s42826-020-00042-4. (**PMID:32382525;PMCID:PMC7203993**) 10.1186/s42826-020-00042-4 PMC7203993 32382525

[CR5] Baker ME (2019) Steroid receptors and vertebrate evolution. Mol Cell Endocrinol 496:110526. 10.1016/j.mce.2019.110526 31376417 10.1016/j.mce.2019.110526

[CR6] Burrows DJ, McGown A, Jain SA, De Felice M, Ramesh TM, Sharrack B, Majid A (2019) Animal models of multiple sclerosis: from rodents to zebrafish. Mult Scler 25(3):306–324. 10.1177/1352458518805246. (**Epub 2018 Oct 15 PMID: 30319015**) 30319015 10.1177/1352458518805246

[CR7] Carney TJ, von der Hardt S, Sonntag C, Amsterdam A, Topczewski J, Hopkins N et al (2007) Inactivation of serine protease Matriptase1a by its inhibitor Hai1 is required for epithelial integrity of the zebrafish epidermis. Development 134:3461–3471 17728346 10.1242/dev.004556

[CR8] Chen H, Lei P, Ji H, Ma J, Fang Y, Yu H, Du J, Qu L, Yang Q, Luo L, Zhang K, Wu W, Jin L, Sun D (2023) Escherichia coli Nissle 1917 ghosts alleviate inflammatory bowel disease in zebrafish. Life Sci 15(329):121956. 10.1016/j.lfs.2023.121956 10.1016/j.lfs.2023.121956 37473802

[CR9] Costa FV, Rosa LV, Quadros VA, Santos ARS, Kalueff AV, Rosemberg DB (2019) Understanding nociception-related phenotypes in adult zebrafish: behavioral and pharmacological characterization using a new acetic acid model. Behav Brain Res 359:570–578. 10.1016/j.bbr.2018.10.009 30296529 10.1016/j.bbr.2018.10.009

[CR10] Costamagna D, Costelli P, Sampaolesi M, Penna F (2015) Role of inflammation in muscle homeostasis and myogenesis. Mediators Inflamm 2015:1–14 10.1155/2015/805172 PMC4609834 26508819

[CR11] Curtright A, Rosser M, Goh S, Keown B, Wagner E, Sharifi J et al (2015) Modeling nociception in zebrafish: a way forward for unbiased analgesic discovery. PLoS ONE 10:e0116766. 10.1371/journal.pone.0116766 25587718 10.1371/journal.pone.0116766 PMC4294643

[CR12] Danilova N, Steiner LA (2002) B cells develop in the zebrafish pancreas. PNAS 99(21):13711–13716. 10.1073/pnas.212515999 12370418 10.1073/pnas.212515999 PMC129751

[CR13] Di Franco G, Usai A, Piccardi M, Cateni P, Palmeri M, Pollina LE, Gaeta R, Marmorino F, Cremolini C, Dente L, Massolo A, Raffa V, Morelli L (2022) Zebrafish patient-derived xenograft model to predict treatment outcomes of colorectal cancer patients. Biomedicines 10(7):1474. 10.3390/biomedicines10071474 35884780 10.3390/biomedicines10071474 PMC9313122

[CR14] Dinarello A, Licciardello G, Fontana CM, Tiso N, Argenton F, Valle LD (2020) Glucocorticoid receptor activities in the zebrafish model: a review. J Endocrinol 247(3):R63–R82. 10.1530/JOE-20-0173 33064662 10.1530/JOE-20-0173

[CR15] El-Gabalawy H, Luenther LC, Bernstein CN (2010) Epidemiology of immune-mediated inflammatory diseases: incidence, prevalence, natural history, and comorbidities. J Rheumatol Suppl 85:2–10. 10.3899/jrheum.091461 20436161 10.3899/jrheum.091461

[CR16] Elks PM, Van Eeden FJ, Dixon G, Wang X, Reyes-Aldasoro CC, Ingham PW, Whyte MKB, Walmsley SR, Renshaw SA (2011) Activation of hypoxia-inducible factor-1α (Hif-1α) delays inflammation resolution by reducing neutrophil apoptosis and reverse migration in a zebrafish inflammation model. Blood 118(3):712–722. 10.1182/blood-2010-12-324186. (**Epub 2011 May 9**) 21555741 10.1182/blood-2010-12-324186

[CR17] Esancy K, Condon L, Feng J, Kimball C, Curtright A, Dhaka A (2018) A zebrafish and mouse model for selective pruritus via direct activation of TRPA1. Elife 21(7):e32036. 10.7554/eLife.32036.PMID:29561265;PMCID:PMC5912907 10.7554/eLife.32036 PMC5912907 29561265

[CR18] Ferrero G, Gomez E, Lyer S, Rovira M, Miserocchi M, Langenau DM, Bertrand JY, Wittamer V (2020) The macrophage-expressed gene (mpeg) 1 identifies a subpopulation of B cells in the adult zebrafish. J Leukocyte Biol. 10.1002/JLB.1A1119-223R 31909502 10.1002/JLB.1A1119-223R PMC7064944

[CR19] Fillatreau S, Six A, Magadan S, Castro R, Sunyer JO, Boudinot P (2013) The astonishing diversity of Ig classes and B cell repertoires in teleost fish. Front Immunol. 10.3389/fimmu.2013.00028 23408183 10.3389/fimmu.2013.00028 PMC3570791

[CR20] Flores E, Dutta S, Bosserman R, van Hoof A, Krachler A-M (2023) Colonization of larval zebrafish (*Danio rerio*) with adherent-invasive *Escherichia coli* prevents recovery of the intestinal mucosa from drug-induced enterocolitis. Msphere. 8(6):e0051223. 10.1128/msphere.00512-23 37971273 10.1128/msphere.00512-23 PMC10732064

[CR21] Frantz WT, Ceol CJ (2022) Research techniques made simple: zebrafish models for human dermatologic disease. J Invest Dermatol 142(3 Pt A):499-506.e1. 10.1016/j.jid.2021.10.016. (**PMID: 35184798**) 35184798 10.1016/j.jid.2021.10.016

[CR22] Gericke J, Harvey BH, Pretorius L, Ollewagen T, Benecke RM, Smith C (2024) Sceletium tortuosum-derived mesembrine significantly contributes to the anxiolytic effect of Zembrin®, but its anti-depressant effect may require synergy of multiple plant constituents. J Ethnopharmacol 319(Pt 1):117113. 10.1016/j.jep.2023.117113(Epub2023Sep1.PMID:37660956) 37660956 10.1016/j.jep.2023.117113

[CR23] Goldstone JV, McArthur AG, Kubota A, Zanette J, Parente T, Jönsson ME, Nelson DR, Stegeman JJ (2010) Identification and developmental expression of the full complement of Cytochrome P450 genes in Zebrafish. BMC Genom 18(11):643. 10.1186/1471-2164-11-643. (**PMID:21087487;PMCID:PMC3012610**) 10.1186/1471-2164-11-643 PMC3012610 21087487

[CR24] Gonzalez-Nunez V, Rodríguez RE (2009) The zebrafish: a model to study the endogenous mechanisms of pain. ILAR J 50(4):373–386. 10.1093/ilar.50.4.373. (**PMID: 19949253**) 19949253 10.1093/ilar.50.4.373

[CR25] Guo L, Dai W, Xu Z, Liang Q, Miller ET, Li S, Gao X, Baldwin MW, Chai R, Li Q (2022) Evolution of brain-expressed biogenic amine receptors into olfactory trace amine-associated receptors. Mol Biol Evol 39(3):msac006. 10.1093/molbev/msac006 35021231 10.1093/molbev/msac006 PMC8890504

[CR26] Häberlein F, Mingardo E, Merten N, Schulze Köhling NK, Reinoß P, Simon K, Japp A, Nagarajan B, Schrage R, Pegurier C, Gillard M, Monk KR, Odermatt B, Kostenis E, Gomeza J (2022) Humanized zebrafish as a tractable tool for in vivo evaluation of pro-myelinating drugs. Cell Chem Biol 29(10):1541-1555.e7. 10.1016/j.chembiol.2022.08.007. (**Epub 2022 Sep 19 PMID: 36126653**) 36126653 10.1016/j.chembiol.2022.08.007

[CR27] Hall CJ, Boyle RH, Sun X, Wicker SM, Misa JP, Krissansen GW, Print CG, Crosier KE, Crosier PS (2014a) Epidermal cells help coordinate leukocyte migration during inflammation through fatty acid-fuelled matrix metalloproteinase production. Nat Commun 23(5):3880. 10.1038/ncomms4880 10.1038/ncomms4880 24852213

[CR28] Hall CJ, Wicker SM, Chien AT, Tromp A, Lawrence LM, Sun X, Krissansen GW, Crosier KE, Crosier PS (2014b) Repositioning drugs for inflammatory disease-fishing for new anti-inflammatory agents. Dis Model Mech 7(9):1069–1081. 10.1242/dmm.016873 25038060 10.1242/dmm.016873 PMC4142727

[CR29] Hammer B, Wagner C, Rankov AD, Reuter S, Bartel S, Hylkema MN, Krüger A, Svanes C, Krauss-Etschmann S (2018) In utero exposure to cigarette smoke and effects across generations: a conference of animals on asthma. Clin Exp Allergy 48(11):1378–1390. 10.1111/cea.13283 30244507 10.1111/cea.13283

[CR30] Hanyang L, Xuanzhe L, Xuyang C, Yujia Q, Jiarong F, Jun S, Zhihua R (2017) Application of zebrafish models in inflammatory bowel disease. Front Immunol 3(8):501. 10.3389/fimmu.2017.00501.PMID:28515725;PMCID:PMC5413514 10.3389/fimmu.2017.00501 PMC5413514 28515725

[CR31] Holdcroft A (2007) Gender bias in research: how does it affect evidence based medicine? J R Soc Med 100(1):2–3. 10.1258/jrsm.100.1.2 17197669 10.1258/jrsm.100.1.2 PMC1761670

[CR32] Howe K, Clark MD, Torroja CF, Torrance J, Berthelot C, Muffato M, Collins JE, Humphray S, McLaren K, Matthews L (2013) The zebrafish reference genome sequence and its relationship to the human genome. Nature 496(7446):498–503. 10.1038/nature12111 23594743 10.1038/nature12111 PMC3703927

[CR33] Huang X, Ai F, Ji C, Tu P, Gao Y, Wu Y, Yan F, Yu T (2021) A rapid screening method of candidate probiotics for inflammatory bowel diseases and the anti-inflammatory effect of the selected strain *Bacillus smithii* XY1. Front Microbiol 17(12):760385. 10.3389/fmicb.2021.760385 10.3389/fmicb.2021.760385 PMC8718878 34975786

[CR34] Itoh T, Matsuda H, Tanioka M, Kuwabara K, Itohara S, Suzuki R (2002) The role of matrix metalloproteinase-2 and matrix metalloproteinase-9 in antibody-induced arthritis. J Immunol 169(5):2643–2647. 10.4049/jimmunol.169.5.2643. (**PMID: 12193736**) 12193736 10.4049/jimmunol.169.5.2643

[CR35] Iwanami N, Hess I, Schorpp M, Boehm T (2017) Chapter 6-Studying the adaptive immune system in zebrafish by transplantation of hematopoietic precursor cells. Methods Cell Biol 138:151–161. 10.1016/bs.mcb.2016.08.003 28129842 10.1016/bs.mcb.2016.08.003

[CR36] Jagannathan-Bogdan M, Zon LI (2013) Hematopoiesis Development 140:2463–2467 23715539 10.1242/dev.083147 PMC3666375

[CR37] Ji J, Hu C, Shao T, Fan D, Zhang N, Lin A, Xiang L, Shao J (2021) Differential immune responses of immunoglobulin Z subclass members in antibacterial immunity in a zebrafish model. Immunology 162(1):105–120. 10.1111/imm.13269. (**Epub 2020 Oct 14**) 32979273 10.1111/imm.13269 PMC7730029

[CR38] Jia HR, Zhu YX, Duan QY, Chen Z, Wu FG (2019) Nanomaterials meet zebrafish: toxicity evaluation and drug delivery applications. J Control Release 311–312:301–318. 10.1016/j.jconrel.2019.08.022. (**Epub 2019 Aug 22 PMID: 31446084**) 31446084 10.1016/j.jconrel.2019.08.022

[CR39] Jomaa B, Hermsen SA, Kessels MY, van den Berg JH, Peijnenburg AA, Aarts JM, Piersma AH, Rietjens IM (2014) Developmental toxicity of thyroid-active compounds in a zebrafish embryotoxicity test. Altex 31(3):303–317. 10.14573/altex.1402011. (**Epub 2014 Apr 10 PMID: 24793664**) 24793664 10.14573/altex.1402011

[CR40] Kamareddine L, Najjar H, Sohail MU, Abdulkader H, Al-Asmakh M (2020) The microbiota and gut-related disorders: insights from animal models. Cells 9(11):2401. 10.3390/cells9112401 33147801 10.3390/cells9112401 PMC7693214

[CR41] Kishi S, Slack B, Uchiyama J, Zhdanovaf IV (2009) Zebrafish as a Genetic Model in Biological and Behavioral Gerontology: Where Development Meets Aging in Vertebrates—A Mini-Review. Gerontology 55(4):430–441. 10.1159/000228892 19654474 10.1159/000228892 PMC2820570

[CR42] Lai Y, Lu Y, Lien H, Huang C, Hwang SL (2016) Foxa2 and Hif1ab regulate maturation of intestinal goblet cells by modulating agr2 expression in zebrafish embryos. Biochem J 473(14):2205–2218. 10.1042/BCJ20160392. (**Epub 2016 May 24**) 27222589 10.1042/BCJ20160392

[CR43] Lam SH, Chua HL, Gong Z, Lam TJ, Sin YM (2004) Development and maturation of the immune system in zebrafish, Danio rerio: a gene expression profiling, in situ hybridization and immunological study. Dev Comp Immunol 28:9–28. 10.1016/s0145-305x(03)00103-4 12962979 10.1016/s0145-305x(03)00103-4

[CR44] Langenau DM, Ferrando AA, Traver D, Kutok JL, Hezel JP, Kanki JP, Zon LI, Look AT, Trede NS (2004) In vivo tracking of T cell development, ablation, and engraftment in transgenic zebrafish. Proc Natl Acad Sci U S A 101(19):7369–7374. 10.1073/pnas.0402248101 15123839 10.1073/pnas.0402248101 PMC409925

[CR45] Lee G, Cheng N, Yu H, Tsai J, Liu T, Wen Z, Chen B, Fu T (2019) A novel zebrafish model to emulate lung injury by folate deficiency-induced swim bladder defectiveness and protease/antiprotease expression imbalance. Sci Rep 9(1):12633. 10.1038/s41598-019-49152-7 31477754 10.1038/s41598-019-49152-7 PMC6718381

[CR46] Leiba J, Özbilgiç R, Hernández L, Demou M, Lutfalla G, Yatime L, Nguyen-Chi M (2023) Molecular actors of inflammation and their signaling pathways: mechanistic insights from zebrafish. Biology (Basel). 12(2):153. 10.3390/biology12020153 36829432 10.3390/biology12020153 PMC9952950

[CR47] Li Y, Miao X, Chen T, Yi X, Wang R, Zhao H, Lee S, Wang X, Zheng Y (2017) Zebrafish as a visual and dynamic model to study the transport of nanosized drug delivery systems across the biological barriers. Colloids Surf, B 156(1):227–235. 10.1016/j.colsurfb.2017.05.022 10.1016/j.colsurfb.2017.05.022 28544957

[CR48] Li J, Sultan Y, Sun Y, Zhang S, Liu Y, Li X (2021) Expression analysis of Hsp90α and cytokines in zebrafish caudal fin regeneration. Dev Comp Immunol 116:103922. 10.1016/j.dci.2020.103922. (**Epub 2020 Nov 11**) 33186559 10.1016/j.dci.2020.103922

[CR49] Lim S, Kang H, Kwon B, Lee JP, Lee J, Choi K (2022) Zebrafish (*Danio rerio*) as a model organism for screening nephrotoxic chemicals and related mechanisms. Ecotoxicol Environ Saf 242:113842. 10.1016/j.ecoenv.2022.113842.(Epub2022Jul8.PMID:35810668) 35810668 10.1016/j.ecoenv.2022.113842

[CR50] Liu X, Li Y, Shinton SA, Rhodes J, Tang L, Feng H, Jette CH, Look AT, Hayakawa K, Hardy RR (2017) Zebrafish B cell development without a Pre-B cell stage, revealed by CD79 fluorescence reporter transgenes. J Immunol 199(5):1706–1715. 10.4049/jimmunol.1700552. (**Epub 2017 Jul 24**) 28739882 10.4049/jimmunol.1700552 PMC5563169

[CR51] Lopez-Luna J, Al-Jubouri Q, Al-Nuaimy W, Sneddon LU (2017) Reduction in activity by noxious chemical stimulation is ameliorated by immersion in analgesic drugs in zebrafish. J Exp Biol 220(Pt 8):1451–1458. 10.1242/jeb.146969. (**PMID: 28424313**) 28424313 10.1242/jeb.146969

[CR52] Maes J, Verlooy L, Buenafe OE, de Witte PA, Esguerra CV, Crawford AD (2012) Evaluation of 14 organic solvents and carriers for screening applications in zebrafish embryos and larvae. PLoS ONE 7(10):e43850. 10.1371/journal.pone.0043850. (**Epub 2012 Oct 17. PMID: 23082109; PMCID: PMC3474771**) 23082109 10.1371/journal.pone.0043850 PMC3474771

[CR53] Martínez-Morcillo FJ, Cantón-Sandoval J, Martínez-Navarro FJ, Cabas I, Martínez-Vicente I, Armistead J, Hatzold J, López-Muñoz A, Martínez-Menchón T, Corbalán-Vélez R, Lacal J, Hammerschmidt M, García-Borrón JC, García-Ayala A, Cayuela ML, Pérez-Oliva AB, García-Moreno D, Mulero V (2021) NAMPT-derived NAD+ fuels PARP1 to promote skin inflammation through parthanatos cell death. PLoS Biol 19(11):e3001455. 10.1371/journal.pbio.3001455. (**eCollection 2021 Nov**) 34748530 10.1371/journal.pbio.3001455 PMC8601609

[CR54] Martínez-Navarro FJ, Martínez-Menchón T, Mulero V, Galindo-Villegas J (2019) Models of human psoriasis: Zebrafish the newly appointed player. Dev Comp Immunol 97:76–87. 10.1016/j.dci.2019.03.018. (**Epub 2019 Apr 4**) 30953679 10.1016/j.dci.2019.03.018

[CR55] Mathias JR, Perrin BJ, Liu TX, Kanki J, Look AT, Huttenlocher A (2006) Resolution of inflammation by retrograde chemotaxis of neutrophils in transgenic zebrafish. J Leukoc Biol 80(6):1281–1288. 10.1189/jlb.0506346. (**Epub 2006 Sep 8 PMID: 16963624**) 16963624 10.1189/jlb.0506346

[CR56] McInnes IB, Gravallese EM (2021) Immune-mediated inflammatory disease therapeutics: past, present and future. Nat Rev Immunol 21:680–686. 10.1038/s41577-021-00603-1 34518662 10.1038/s41577-021-00603-1 PMC8436867

[CR57] Moss JJ, Wirth M, Tooze SA, Lane JD, Hammond CL (2021) Autophagy coordinates chondrocyte development and early joint formation in zebrafish. FASEB J 35(11):e22002. 10.1096/fj.202101167R.PMID:34708458;PMCID:PMC9670821 34708458 10.1096/fj.202101167R PMC9670821

[CR58] Muire PJ, Hanson LA, Wills R, Petrie-Hanson L (2017) Differential gene expression following TLR stimulation in *rag1*^*-/-*^ mutant zebrafish tissues and morphological descriptions of lymphocyte-like cell populations. PLoS ONE 12(9):e0184077. 10.1371/journal.pone.0184077 28910320 10.1371/journal.pone.0184077 PMC5598945

[CR59] Ni Y, Zhang Y, Zheng L, Rong N, Yang Y, Gong P, Yang Y, Siwu X, Zhang C, Zhu L, Fu Z (2023) Bifidobacterium and Lactobacillus improve inflammatory bowel disease in zebrafish of different ages by regulating the intestinal mucosal barrier and microbiota. Life Sci 1(324):121699. 10.1016/j.lfs.2023.121699 10.1016/j.lfs.2023.121699 37061125

[CR60] Ohnesorge N, Heinl C, Lewejohann L (2021) Current methods to investigate nociception and pain in zebrafish. Front Neurosci 8(15):632634. 10.3389/fnins.2021.632634. (**PMID:33897350;PMCID:PMC8061727**) 10.3389/fnins.2021.632634 PMC8061727 33897350

[CR61] Ollewagen T, Myburgh KH, van de Vyver M, Smith C (2021a) Rheumatoid cachexia: the underappreciated role of myoblast, macrophage and fibroblast interplay in the skeletal muscle niche. J Biomed Sci 28(1):15. 10.1186/s12929-021-00714-w. (**PMID:33658022;PMCID:PMC7931607**) 33658022 10.1186/s12929-021-00714-w PMC7931607

[CR62] Ollewagen T, Powrie YSL, Myburgh KH, Smith C (2021b) Unresolved intramuscular inflammation, not diminished skeletal muscle regenerative capacity, is at the root of rheumatoid cachexia: insights from a rat CIA model. Physiol Rep 9(22):e15119. 10.14814/phy2.15119 34806343 10.14814/phy2.15119 PMC8606867

[CR63] Ollewagen T, Benecke RM, Smith C (2023) High species homology potentiates quantitative inflammation profiling in zebrafish using immunofluorescence. Heliyon. 10(1):e23635. 10.1016/j.heliyon.2023.e23635. (**eCollection 2024 Jan 15**) 38187273 10.1016/j.heliyon.2023.e23635 PMC10770569

[CR64] Paik H, Chung A, Park H, Park RW, Suk K, Kim J, Kim H, Lee K, Butte AJ (2015) Repurpose terbutaline sulfate for amyotrophic lateral sclerosis using electronic medical records. Sci Rep 5(5):8580. 10.1038/srep08580 25739475 10.1038/srep08580 PMC4894399

[CR65] Pan Y, Tong S, Hsu C, Weng J, Chung B (2022) Zebrafish establish female germ cell identity by advancing cell proliferation and meiosis. Front Cell Dev Biol. 10:866267. 10.3389/fcell.2022.866267. (**eCollection 2022**) 35445010 10.3389/fcell.2022.866267 PMC9013747

[CR66] Patton EE, Tobin DM (2019) Spotlight on zebrafish: the next wave of translational research. Dis Model Mech 12(3):dmm039370. 10.1242/dmm.039370 30858282 10.1242/dmm.039370 PMC6451428

[CR67] Patton EE, Zon LI, Langenau DM (2021) Zebrafish disease models in drug discovery: from preclinical modelling to clinical trials. Nat Rev Drug Discov 20(8):611–628. 10.1038/s41573-021-00210-8 34117457 10.1038/s41573-021-00210-8 PMC9210578

[CR68] Paul CD, Devine A, Bishop K et al (2019) Human macrophages survive and adopt activated genotypes in living zebrafish. Sci Rep 9:1759. 10.1038/s41598-018-38186-y 30741975 10.1038/s41598-018-38186-y PMC6370805

[CR69] Poon KL, Wang X, Ng AS, Goh WH, McGinnis C, Fowler S, Carney TJ, Wang H, Ingham PW (2017) Humanizing the zebrafish liver shifts drug metabolic profiles and improves pharmacokinetics of CYP3A4 substrates. Arch Toxicol 91(3):1187–1197. 10.1007/s00204-016-1789-5. (**Epub 2016 Aug 2 PMID: 27485346**) 27485346 10.1007/s00204-016-1789-5

[CR70] Porter BA, Mueller T (2020) The zebrafish amygdaloid complex—functional ground plan, molecular delineation, and everted topology. Front Neurosci. 10.3389/fnins.2020.00608 32765204 10.3389/fnins.2020.00608 PMC7378821

[CR71] Powrie YSL, Smith C (2018) Central intracrine DHEA synthesis in ageing-related neuroinflammation and neurodegeneration: therapeutic potential? J Neuroinflammation 15(1):289. 10.1186/s12974-018-1324-0 30326923 10.1186/s12974-018-1324-0 PMC6192186

[CR72] Powrie YSL, Strydom M, Aucamp M, Schellack N, Steenkamp V, Smith C (2022) Zebrafish behavioral response to ivermectin: insights into potential neurological risk. Medicine in Drug Discovery 16:100141. 10.1016/j.medidd.2022.100141

[CR73] Pretorius L, Smith C (2023a) Tyramine-induced gastrointestinal dysregulation is attenuated via estradiol associated mechanisms in a zebrafish larval model. Toxicol Appl Pharmacol 461:116399. 10.1016/j.taap.2023.116399.(Epub2023Jan27) 36716863 10.1016/j.taap.2023.116399

[CR74] Pretorius L, Smith C (2023b) Translation of preclinical ethnomedicine data in LMICs: the example of rooibos. Front Pharmacol 19(14):1328828. 10.3389/fphar.2023.1328828 10.3389/fphar.2023.1328828 PMC10763253 38174224

[CR75] Pretorius L, Smith C (2024) Green rooibos (*Aspalathus linearis*) promotes gut health: insight into mechanisms. J Ethnopharmacol 319(Pt 3):117379. 10.1016/j.jep.2023.117379. (**Epub 2023 Nov 2**) 37923252 10.1016/j.jep.2023.117379

[CR76] Progatzky F, Cook HT, Lamb JR, Bugeon L, Dallman MJ (2016) Mucosal inflammation at the respiratory interface: a zebrafish model. Am J Physiol Lung Cell Mol Physiol 310(6):L551–L561. 10.1152/ajplung.00323.2015. (**Epub 2015 Dec 30**) 26719149 10.1152/ajplung.00323.2015 PMC4796261

[CR77] Quiñonez-Silvero C, Hübner K, Herzog W (2020) Development of the brain vasculature and the blood-brain barrier in zebrafish. Dev Biol 457(2):181–190. 10.1016/j.ydbio.2019.03.005 30862465 10.1016/j.ydbio.2019.03.005

[CR78] Robertson GN, Croll RP, Smith FM (2014) The structure of the caudal wall of the zebrafish (Danio rerio) swim bladder: evidence of localized lamellar body secretion and a proximate neural plexus. J Morphol 275(8):933–948. 10.1002/jmor.20274. (**Epub 2014 Mar 19**) 24643973 10.1002/jmor.20274

[CR79] Roth GA, GBD 2017 Causes of Death Collaborators (2018) Global, regional, and national age-sex-specific mortality for 282 causes of death in 195 countries and territories, 1980–2017: a systematic analysis for the Global Burden of Disease Study 2017. Lancet 392(10159):1736–1788. 10.1016/S0140-6736(18)32203-7. (**Epub 2018 Nov 8. Erratum in: Lancet. 2019 Jun 22;393(10190):e44. Erratum in: Lancet. 2018 Nov 17;392(10160):2170. PMID: 30496103; PMCID: PMC6227606**) 30496103 10.1016/S0140-6736(18)32203-7 PMC6227606

[CR80] Schaaf MJ, Champagne D, van Laanen IH, van Wijk DC, Meijer AH, Meijer OC, Spaink HP, Richardson MK (2008) Discovery of a functional glucocorticoid receptor beta-isoform in zebrafish. Endocrinology 149(4):1591–1599. 10.1210/en.2007-1364 18096659 10.1210/en.2007-1364

[CR81] Schoonheim PJ, Chatzopoulou A, Schaaf MJM (2010) The zebrafish as an in vivo model system for glucocorticoid resistance. Steroids 75(12):918–925. 10.1016/j.steroids.2010.05.010. (**Epub 2010 May 21**) 20493895 10.1016/j.steroids.2010.05.010

[CR82] Schork N (2015) Personalized medicine: Time for one-person trials. Nature 520:609–611. 10.1038/520609a 25925459 10.1038/520609a

[CR83] Schroeder PG, Sneddon LU (2017) Exploring the efficacy of immersion analgesics in zebrafish using an integrative approach. Appl Anim Behav Sci 187:93–102. 10.1016/j.applanim.2016.12.003

[CR84] Seyhan AA (2019) Lost in translation: the valley of death across preclinical and clinical divide—identification of problems and overcoming obstacles. Transl Med Commun 4:18. 10.1186/s41231-019-0050-7

[CR85] Smits DJ, Dekker J, Schot R, Tabarki B, Alhashem A, Demmers JAA, Dekkers DHW, Romito A, Van der Spek PJ, Van Ham TJ, Avella AM, Mancini GMS (2023) CLEC16A interacts with retromer and TRIM27, and its loss impairs endosomal trafficking and neurodevelopment. Hum Genet 142:379–397. 10.1007/s00439-022-02511-3 36538041 10.1007/s00439-022-02511-3 PMC9950183

[CR86] Sneddon LU (2019) Evolution of nociception and pain: evidence from fish models. Philos Trans R Soc 374:20190290. 10.1098/rstb.2019.0290 10.1098/rstb.2019.0290 PMC6790376 31544617

[CR87] Sternberg EM (2001) Neuroendocrine regulation of autoimmune/inflammatory disease. J Endocrinol 169(3):429–435. 10.1677/joe.0.1690429 11375112 10.1677/joe.0.1690429

[CR88] Strynatka KA, Gurrola-Gal MC, Berman JN, McMaster CR (2018) How surrogate and chemical genetics in model organisms can suggest therapies for human genetic diseases. Genetics 208:833–851. 10.1534/genetics.117.300124 29487144 10.1534/genetics.117.300124 PMC5844338

[CR89] Sun A, Gao W, Hu H, Zhoub S (2022) Why 90% of clinical drug development fails and how to improve it? Acta Pharm Sin b 12(7):3049–3062. 10.1016/j.apsb.2022.02.002 35865092 10.1016/j.apsb.2022.02.002 PMC9293739

[CR90] Sundberg JP, Elson CO, Bedigian H, Birkenmeier EH (1994) Spontaneous, heritable colitis in a new substrain of C3H/HeJ mice. Gastroenterology 107:1726–1735. 10.1016/0016-5085(94)90813-323 7958684 10.1016/0016-5085(94)90813-3

[CR91] Sunke R, Bankala R, Thirupataiah B, Ramarao EVVS, Kumar JS, Doss HM, Medishetti R, Kulkarni P, Kapavarapu RK, Rasool M, Mudgal J, Mathew JE, Shenoy GG, Parsa KVL, Pal M (2019) InCl3 mediated heteroarylation of indoles and their derivatization via CH activation strategy: Discovery of 2-(1H-indol-3-yl)-quinoxaline derivatives as a new class of PDE4B selective inhibitors for arthritis and/or multiple sclerosis. Eur J Med Chem 174:198–215. 10.1016/j.ejmech.2019.04.020 31035240 10.1016/j.ejmech.2019.04.020

[CR92] Trede NS, Langenau DM, Traver D, Look T, Zon LI (2004) The use of zebrafish to understand immunity. Immunity 20(4):367–379. 10.1016/s1074-7613(04)00084-6 15084267 10.1016/s1074-7613(04)00084-6

[CR93] Trede NS, Heaton W, Ridges S, Sofla H, Cusick M, Bearss D, Jones D, Fujinami RS (2013) Discovery of biologically active oncologic and immunologic small molecule therapies using zebrafish: overview and example of modulation of T cell activation. Curr Protoc Pharmacol. 10.1002/0471141755.ph1424s60 23456612 10.1002/0471141755.ph1424s60

[CR94] Uller L, Persson CGA, Erjefält JS (2006) Resolution of airway disease: removal of inflammatory cells through apoptosis, egression or both? Trends Pharmacol Sci 27(9):461–466. 10.1016/j.tips.2006.07.006. (**Epub 2006 Jul 31**) 16876880 10.1016/j.tips.2006.07.006

[CR95] Usai A, Di Franco G, Piccardi M, Cateni P, Pollina LE, Vivaldi C, Vasile E, Funel N, Palmeri M, Dente L, Falcone A, Giunchi D, Massolo A, Raffa V, Morelli L (2021) Zebrafish patient-derived xenografts identify chemo-response in pancreatic ductal adenocarcinoma patients. Cancers (basel) 13(16):4131. 10.3390/cancers13164131 34439284 10.3390/cancers13164131 PMC8394309

[CR96] Vomero M, Barbati C, Colasanti T, Perricone C, Novelli L, Ceccarelli F, Spinelli FR, Di Franco M, Conti F, Valesini G, Alessandri C (2018) Autophagy and rheumatoid arthritis: current knowledges and future perspectives. Front Immunol 18(9):1577. 10.3389/fimmu.2018.01577.PMID:30072986;PMCID:PMC6058034 10.3389/fimmu.2018.01577 PMC6058034 30072986

[CR97] Wcisel DJ, Dornburg A, McConnell SC, Hernandez KM, Andrade J, de Jong JLO, Litman GW, Yoder JA (2023) A highly diverse set of novel immunoglobulin-like transcript (NILT) genes in zebrafish indicates a wide range of functions with complex relationships to mammalian receptors. Immunogenetics 75(1):53–69. 10.1007/s00251-022-01270-9. (**Epub 2022 Jul 23**) 35869336 10.1007/s00251-022-01270-9 PMC9845131

[CR98] Webb AE, Driever W, Kimelman D (2008) psoriasis regulates epidermal development in zebrafish. Dev Dyn 237:1153–1164 18351656 10.1002/dvdy.21509

[CR99] Wen J, Weng J, Lu W, Tao X, Cheng H, Tang Y (2023) The expression of plasmacytoid dendritic cells and TLR7/9-MyD88-IRAKs pathway in chronic eczema lesions. Clin Cosmet Investig Dermatol 24(16):1079–1087. 10.2147/CCID.S405491. (**PMID:37123625;PMCID:PMC10145378**) 10.2147/CCID.S405491 PMC10145378 37123625

[CR100] Winata CL, Korzh S, Kondrychyn I, Zheng WL, Korzh V, Gong ZY (2009) Development of zebrafish swimbladder: the requirement of Hedgehog signaling in specification and organization of the three tissue layers. Dev Biol 331:222–236 19422819 10.1016/j.ydbio.2009.04.035

[CR101] Wu D, GBD 2019 IMID Collaborators (2023) Global, regional, and national incidence of six major immune-mediated inflammatory diseases: findings from the global burden of disease study 2019. EClinicalMedicine 64:102193. 10.1016/j.eclinm.2023.102193. (**eCollection 2023 Oct**) 37731935 10.1016/j.eclinm.2023.102193 PMC10507198

[CR102] Wyatt RA, Crawford BD (2021) Post-translational activation of Mmp2 correlates with patterns of active collagen degradation during the development of the zebrafish tail. Dev Biol 477:155–163. 10.1016/j.ydbio.2021.05.016. (**Epub 2021 May 28 PMID: 34058190**) 34058190 10.1016/j.ydbio.2021.05.016

[CR103] Xia H, Chen H, Cheng X, Yin M, Yao X, Ma J, Huang M, Chen G, Liu H (2022) Zebrafish: an efficient vertebrate model for understanding role of gut microbiota. Mol Med 28(1):161. 10.1186/s10020-022-00579-1 36564702 10.1186/s10020-022-00579-1 PMC9789649

[CR104] Xie Y, Meijer AH, Schaaf MJM (2021) Modeling Inflammation in Zebrafish for the Development of Anti-inflammatory Drugs. Front Cell Dev Biol 15(8):620984. 10.3389/fcell.2020.620984. (**eCollection2020**) 10.3389/fcell.2020.620984 PMC7843790 33520995

[CR105] Xu F, Yang F, Qiu Y, Wang C, Zou Q, Wang L, Li X, Jin M, Liu K, Zhang S, Zhang Y, Li B (2024) The alleviative effect of C-phycocyanin peptides against TNBS-induced inflammatory bowel disease in zebrafish via the MAPK/Nrf2 signaling pathways. Fish Shellfish Immunol 145:109351. 10.1016/j.fsi.2023.109351 38171429 10.1016/j.fsi.2023.109351

[CR106] Yang Y, Tomkovich S, Jobin C (2014) Could a swimming creature inform us on intestinal diseases? Lessons from Zebrafish. Inflamm Bowel Dis 20:956–966. 10.1097/01.MIB.0000442923.85569.68 24577115 10.1097/01.MIB.0000442923.85569.68 PMC4635025

[CR107] Yao Y, Sun S, Fei F, Wang J, Wang Y, Zhang R, Wu J, Liu L, Liu X, Cui Z, Li Q, Yu M, Dang Y, Wang X (2017) Screening in larval zebrafish reveals tissue-specific distribution of fifteen fluorescent compounds. Dis Model Mech 10(9):1155–1164. 10.1242/dmm.028811. (**Epub 2017 Jul 28. PMID: 28754836; PMCID: PMC5611963**) 28754836 10.1242/dmm.028811 PMC5611963

[CR108] Ye B, Deng X, Shao L, Lu Y, Xiao R, Liu Y, Jin Y, Xie Y, Zhao Y, Luo L, Ma S, Gao M, Zhang L, He J, Zhang W, Chen Y, Xia C, Deng M, Liu T, Zhao Q, Chen S, Chen Z (2015) Vibsanin B preferentially targets HSP90β, inhibits interstitial leukocyte migration, and ameliorates experimental autoimmune encephalomyelitis. J Immunol 194(9):4489–4497. 10.4049/jimmunol.1402798 25810397 10.4049/jimmunol.1402798

[CR109] Yu Y, Chen J, Zhang X, Wang Y, Wang S, Zhao L, Wang Y (2021) Identification of anti-inflammatory compounds from Zhongjing formulae by knowledge mining and high-content screening in a zebrafish model of inflammatory bowel diseases. Chin Med 16(1):42. 10.1186/s13020-021-00452-z 34059101 10.1186/s13020-021-00452-z PMC8166029

[CR110] Zhao Q, Chang H, Zheng J, Li P, Ye L, Pan R, Li D, Shao J, Zhao JC, Chen Y (2023) A novel Trmt5-deficient zebrafish model with spontaneous inflammatory bowel disease-like phenotype. Signal Transduct Target Ther 8:86. 10.1038/s41392-023-01318-6 36849517 10.1038/s41392-023-01318-6 PMC9971238

[CR111] Zheng W, Wang Z, Collins JE, Andrews RM, Stemple D (2011) Comparative transcriptome analyses indicate molecular homology of zebrafish swim bladder and mammalian lung. PLoS ONE 6(8):e24019. 10.1371/journal.pone.0024019 21887364 10.1371/journal.pone.0024019 PMC3162596

[CR112] Zhong X, Li J, Lu F, Zhang J, Guo L (2022) Application of zebrafish in the study of the gut microbiome. Animal Model Exp Med 5(4):323–336. 10.1002/ame2.12227. (**Epub 2022 Apr 12**) 35415967 10.1002/ame2.12227 PMC9434591

